# Reframing Antimicrobial Peptides beyond Direct Antimicrobial Activity toward Host-Directed Functions and Disease-Specific Therapeutic Applications

**DOI:** 10.4014/jmb.2606.06034

**Published:** 2026-09-02

**Authors:** Minho Lee

**Affiliations:** 1 Department of Microbiology, College of Medicine, Hallym University, Chuncheon 24252, Republic of Korea; 2 Institute of Medical Science, College of Medicine, Hallym University, Chuncheon 24252, Republic of Korea

**Keywords:** Antimicrobial peptides, Host–microbe interface, Biofilm, Peptide engineering, Immunomodulation, Nanomedicine

## Abstract

Antimicrobial peptides (AMPs) have been extensively investigated as alternatives to conventional antibiotics to combat multidrug-resistant bacteria. This narrow antibiotic-replacement framework underestimates the biological and translational potential of AMPs. The present review reframes them as disease-context-dependent therapeutic platforms rather than universal antibiotic substitutes. In particular, this review critically examines their microbiological basis, mechanisms of action, emerging applications, engineering strategies, formulation approaches, translational limitations, and criteria for translational development. AMPs exert diverse activities at the host–microbe interface. These effects include direct antimicrobial activity, membrane perturbation, intracellular targeting, antibiofilm effects, endotoxin neutralization, immunomodulation, and tissue repair. Hence, these multifunctional properties support their potential as therapeutic candidates for chronic wounds, biofilm-associated infections, device-related infections, mucosal and respiratory disorders, and inflammation-associated disorders. Furthermore, recent advances in peptide engineering, artificial intelligence-guided design, and delivery technologies, including nanocarriers, hydrogels, and surface immobilization, have expanded the design space and translational opportunities for AMPs. Major translational barriers remain, including cytotoxicity, hemolysis, proteolytic instability, resistance selection and cross-resistance, limited pharmacokinetic and pharmacodynamic characterization, manufacturing costs, scale-up challenges, and uncertain clinical positioning. Overall, AMPs should be developed through a translational decision matrix that integrates mechanism, microbial susceptibility, host response, formulation, safety, and disease-relevant efficacy rather than through antimicrobial potency ranking alone.

## Introduction

Antimicrobial resistance (AMR) is a major threat to modern medicine [1, 2]. In 2019, bacterial AMR was associated with 4.95 million deaths and directly attributable to approximately 1.27 million deaths worldwide; annual attributable deaths are projected to reach 1.91 million by 2050 [1, 2]. This burden has intensified interest in antimicrobial strategies for resistant pathogens and infection settings inadequately addressed by conventional antibiotics [3, 4].

**Fig. 1:**
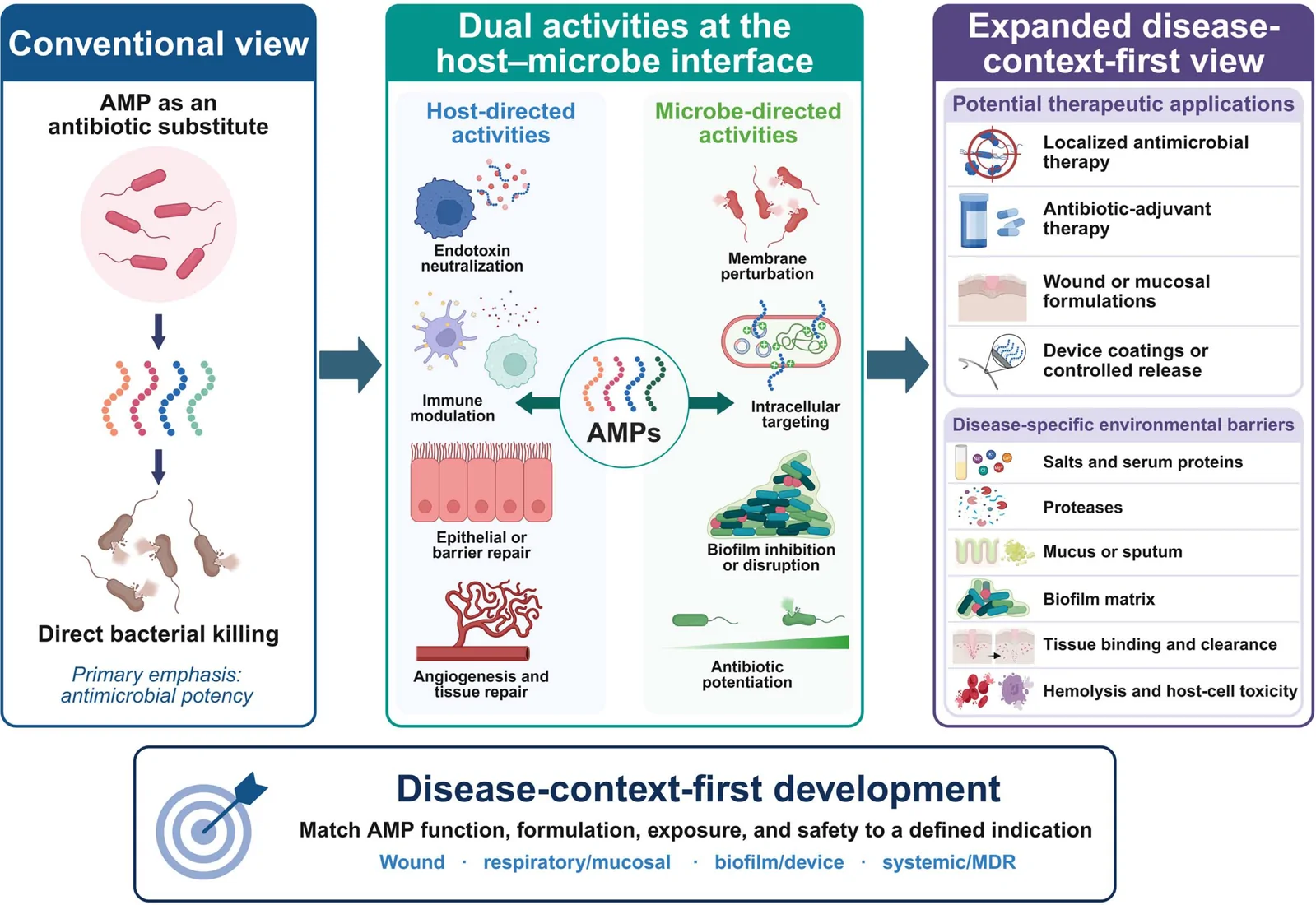
Conceptual progression from the conventional antibiotic-substitute view of antimicrobial peptides to disease-context-first multifunctional therapeutic development. The left panel illustrates the conventional view of antimicrobial peptides (AMPs) as substitutes for conventional antibiotics whose principal function is direct bacterial killing. The middle panel expands this view by separating the complementary activities of AMPs at the host–microbe interface. Microbe-directed activities include membrane perturbation, intracellular targeting, biofilm inhibition or disruption, and antibiotic potentiation, whereas host-directed activities include endotoxin neutralization, immune modulation, epithelial or barrier repair, and angiogenesis and tissue repair. The right panel presents the expanded disease-context-first framework. Potential therapeutic applications include localized antimicrobial therapy, antibiotic-adjuvant strategies, wound or mucosal formulations, and device coatings or controlled-release systems. Their performance is conditioned by disease-specific environmental and translational barriers, including salts and serum proteins, proteases, mucus or sputum, biofilm matrices, tissue binding and clearance, and hemolysis and host-cell toxicity. The bottom panel summarizes alignment of AMP function, formulation, exposure, safety, and indication rather than selecting candidates on the basis of direct antibacterial potency alone. This figure was conceptualized by the author and created using BioRender.com.

Antimicrobial peptides (AMPs) are evolutionarily conserved components of innate defense that can act through mechanisms distinct from those of many conventional antibiotics [3, 4]. Although AMPs have often been framed as antibiotic substitutes, this view does not capture their broader activities at the host–microbe interface. As summarized in Fig. 1, AMP functions extend beyond direct bacterial killing to microbe-directed activities, including membrane perturbation, intracellular targeting, biofilm inhibition or disruption, and antibiotic potentiation. They also include host-directed activities such as endotoxin neutralization, immune modulation, epithelial or barrier repair, and angiogenesis and tissue repair [3, 4].

Representative studies illustrate this functional breadth. The antibiofilm peptide 1018 potentiated several antibiotics against biofilms formed by multidrug-resistant pathogens, whereas LL-37-loaded poly(lactic-co-glycolic acid) nanoparticles showed antimicrobial activity *in vitro* and accelerated wound healing *in vivo* [5, 6]. Together with broader evidence, these studies support localized antimicrobial treatment, antibiotic-adjuvant strategies, wound or mucosal formulations, and device-associated applications [3, 4, 7]. AMP performance depends on disease-specific conditions such as salts and serum proteins, proteases, mucus or sputum, biofilm matrices, tissue binding and clearance, and host-cell toxicity [4, 8]. These factors can alter free active peptide concentration, target accessibility, and host compatibility; therefore, activity measured under standardized planktonic conditions may not predict performance at the intended site of use [8].

Advances in peptide engineering, artificial intelligence (AI)-guided discovery, and formulation are expanding the design space for AMP therapeutics. Chemical modification, D-amino acid substitution, cyclization, lipidation, peptidomimetics, and delivery systems can improve stability, exposure, and pharmacological performance [3, 8]. Recent explainable deep-learning and generative approaches have identified active AMP candidates against multidrug-resistant pathogens in experimental models [9, 10]. Nonetheless, cytotoxicity, hemolysis, proteolysis, serum binding, uncertain pharmacokinetic/pharmacodynamic (PK/PD) behavior, manufacturing constraints, and uncertain clinical positioning remain major translational barriers [8]. These liabilities argue against ranking candidates by antimicrobial potency alone and favor strategies that define the required function and exposure for a specific therapeutic context.

In this review, I define a disease-context-first framework as an indication-led strategy in which the intended clinical use and target product profile are specified before AMP lead selection [11]. The unmet need, target pathogen or microbial community, planktonic or biofilm physiology, tissue compartment, host inflammatory or repair state, and local environmental constraints are then translated into predefined requirements for peptide function, delivery, exposure, safety, and disease-relevant efficacy [12]. This differs from an activity-led, candidate-first workflow that initially prioritizes broad-spectrum activity or low minimum inhibitory concentration (MIC) values and addresses formulation and clinical positioning later [12]. In a disease-context-first strategy, the indication-specific product profile instead guides discovery, engineering, formulation, and go/no-go criteria from the outset. The framework does not restrict a peptide to one indication; rather, each candidate–formulation combination should be evaluated against the requirements of a predefined use. For example, wound-directed candidates may prioritize local exposure and tissue repair, whereas device coatings may prioritize anti-adhesion activity and cytocompatibility (Table 1) [6, 7].

The distinctive contribution of this review is to integrate microbial physiology, host responses, disease microenvironments, engineering, delivery, safety, resistance risk, microbiome effects, and disease-relevant validation within a unified translational framework. Existing reviews have addressed many of these domains individually [3, 4, 8]; the present review connects them through indication-led decision making and explicit preclinical go/no-go criteria. This perspective shifts the central question from whether AMPs can replace antibiotics to which AMP functions and delivery strategies are appropriate for a defined indication. Accordingly, the review examines AMPs as multifunctional therapeutic platforms whose development should be guided by indication-specific biological requirements and clinically relevant validation rather than antimicrobial potency alone.

### Molecular and Microbiological Basis of AMP Application

**Natural AMP families and engineered scaffolds.** AMPs comprise a structurally and functionally diverse group of natural and engineered molecules rather than a single mechanistic class [3, 4]. Natural AMPs occur in bacteria, fungi, plants, insects, marine organisms, and vertebrates, where they contribute to microbial competition, innate defense, epithelial protection, and host–microbe communication [3, 13-17]. Biological origin provides useful context for biosynthesis and ecological function but does not by itself determine antimicrobial spectrum, mechanism, host-cell selectivity, or translational suitability.

Representative peptides illustrate this diversity. Human β-defensin 3 is a highly cationic, cysteine-rich vertebrate peptide with broad activity against Gram-positive and Gram-negative bacteria and fungi and can promote monocyte chemotaxis [18]. The human cathelicidin LL-37 combines antimicrobial, endotoxin-neutralizing, immunomodulatory, and epithelial-repair activities [4, 19, 20], including promotion of keratinocyte migration through epidermal growth factor receptor transactivation [21]. Plectasin, a fungal defensin from *Pseudoplectania nigrella*, is active against *Streptococcus pneumoniae*, including resistant isolates, and showed efficacy in murine infection models [22]. Among bacterial bacteriocins, nisin binds the cell-wall precursor lipid II and uses it as a docking molecule for membrane-pore formation [14, 23]. Cecropin A, a linear amphipathic insect peptide, kills *Escherichia coli* in association with membrane permeabilization and depolarization [24]. Marine piscidin adopts an amphipathic α-helical conformation in membrane-mimetic environments and permeabilizes bilayers through a mechanism consistent with toroidal-pore formation [16, 25]. The plant cyclotide cycloviolacin O2 is bactericidal against several Gram-negative species, and modification of surface-exposed charged residues reduces antibacterial activity [17, 26]. These examples emphasize that therapeutic behavior depends more directly on sequence, structure, mechanism, and exposure context than on biological source alone.

Natural scaffolds also require engineering because they are rarely optimized for human pharmacology, manufacturability, toxicity, or disease-specific delivery [3, 27]. Cyclization, D-amino acid substitution, α-helical stapling, lipidation, and peptidomimetic or synthetic amphiphilic scaffolds can improve protease resistance, conformational stability, or membrane association [27-33]. The same modifications may alter aggregation, serum binding, tissue distribution, immunogenicity, and host-cell toxicity. Engineered analogues should be evaluated as distinct molecular entities rather than assumed to retain the biological profile of their parent peptides. Representative families and engineered scaffolds are summarized in Supplementary Table S1.

**Mechanistic structure–activity–toxicity relationships.** AMP activity emerges from the combined effects of sequence, net charge and charge distribution, amphipathicity, hydrophobicity, conformational propensity, length, and self-association [3, 34-36]. For many cationic AMPs, electrostatic attraction to anionic microbial components increases the local peptide concentration at the cell surface, but binding alone does not ensure membrane insertion or killing. Amphipathicity favors orientation at the membrane–water interface, whereas hydrophobicity promotes deeper bilayer partitioning. Membrane-induced α-helical or β-sheet formation, peptide length, flexibility, and oligomerization further influence whether a peptide remains surface-bound, inserts into the bilayer, spans the membrane, or forms higher-order assemblies [3, 28, 35-37].

Membrane disruption generally becomes more pronounced after interfacial peptide accumulation reaches a threshold local peptide-to-lipid ratio, producing lipid-packing defects, curvature stress, transient aqueous defects, leakage, depolarization, or membrane disintegration [31, 35, 38, 39]. Barrel-stave, toroidal-pore, and carpet-like models are useful descriptions but should not be treated as rigid or mutually exclusive mechanisms. Barrel-stave pores are predominantly peptide-lined, toroidal pores are lined by both peptides and continuously curved lipid headgroups, and carpet-like mechanisms arise when surface-associated peptides collectively destabilize the bilayer above a concentration threshold. Magainin 2, for example, couples pore formation with phospholipid flip-flop and peptide translocation [38], whereas other cationic peptides can reorganize membranes and delocalize peripheral membrane proteins without forming stable uniform pores [39]. Membrane activity is better viewed as a dynamic continuum than assigned automatically to a single canonical model.

Selectivity reflects differential association and partitioning between microbial and host membranes. Bacterial envelopes generally expose a higher density of anionic lipids and polymers than the outer leaflet of mammalian plasma membranes, which is largely zwitterionic and cholesterol-rich [35, 36]. Selectivity nevertheless depends on hydrophobicity, hydrophobic moment, secondary structure, aggregation, membrane fluidity, membrane potential, and peptide concentration as well as charge [36]. Excessive hydrophobicity, constitutive helicity, or self-association can increase hemolysis, epithelial toxicity, serum binding, or nonspecific tissue association. A selectivity index can compare antimicrobial activity with a defined host-toxicity threshold among related analogues tested under matched conditions, and rational sequence design can improve this balance [36, 37]. MIC-based ratios are assay-dependent and do not capture bactericidal kinetics, biofilm activity, resistance selection, biological-matrix effects, formulation-dependent exposure, or toxicity in relevant host cells [3, 8]. Selectivity is best interpreted within an integrated efficacy–safety assessment rather than as a standalone developmental criterion.

Membrane interaction can also provide entry to intracellular targets rather than constitute the terminal lethal event. Some AMPs translocate without extensive membrane disruption and subsequently engage intracellular macromolecules [30, 40-42]. Buforin II can penetrate microbial membranes with limited membrane damage and inhibit cellular functions [40], whereas the proline-rich peptide Onc112 binds the bacterial ribosome and blocks the translation-initiation complex [41]. Other non-lytic AMPs can interfere with nucleic acids, protein folding, cell division, cell-wall biosynthesis, or enzymes [43]. Intracellular potency depends not only on target affinity but also on transport across the bacterial envelope, intracellular stability, and avoidance of extracellular or periplasmic sequestration.

These structure–mechanism relationships are conditional rather than universal. The same peptide may remain predominantly surface-bound at a low concentration, produce transient permeabilization at intermediate levels, and cause extensive disruption at higher concentrations. Its behavior can also change with envelope architecture, membrane potential, bacterial growth state, ionic strength, pH, serum proteins, proteases, mucus, biofilm matrix, and formulation [3, 8, 34, 36, 38-41, 44]. Model membranes are valuable for isolating biophysical phenomena but do not reproduce the complexity of intact bacterial envelopes. Accordingly, dye leakage, circular dichroism, or membrane-depolarization measurements alone cannot establish the lethal mechanism. Mechanistic assignment should integrate membrane binding and permeability, membrane-potential measurements, leakage, microscopy, peptide translocation, intracellular target engagement, killing kinetics, and host-cell toxicity. Structure–mechanism relationships are best treated as experimentally testable, context-dependent hypotheses rather than fixed properties inferred solely from sequence or secondary structure.

### Microbiological Determinants of AMP Susceptibility

**Bacterial envelope architecture and adaptive responses.** Bacterial envelope architecture is a major determinant of AMP susceptibility. In Gram-positive bacteria, peptidoglycan and wall or lipoteichoic acids can alter peptide accumulation, diffusion, and membrane access [45, 46]. In Gram-negative bacteria, the outer membrane, LPS, lipid A, capsule, and periplasm create additional permeability barriers that affect peptide binding, uptake, and access to inner-membrane targets [47, 48]. These differences contribute to species- and physiological-state-dependent variation in AMP potency [3, 34].

Bacteria can further reduce susceptibility through surface remodeling and other adaptive responses [34, 49]. D-alanylation of teichoic acids, MprF-mediated lysinylation of phosphatidylglycerol, and lipid A modification can decrease electrostatic peptide association or penetration [34, 46, 47, 50]. Proteolytic inactivation, extracellular sequestration, efflux, capsule-mediated shielding, and envelope stress responses provide additional protection [34, 49]. Two-component regulatory systems coordinate many of these changes by remodeling surface charge, membrane composition, and peptide transport (Table 2) [34, 47, 49].

**Biofilm-associated AMP tolerance and resistance.** Biofilms add community-level tolerance that should be distinguished from stable genetic resistance [44, 51]. Their extracellular matrix can restrict diffusion or sequester cationic peptides, while oxygen, nutrient, and pH gradients generate heterogeneous physiological states [44, 51]. Slow growth, stress responses, and persister-like subpopulations can reduce killing even when planktonic MIC values indicate susceptibility [51, 52]. These effects help explain why planktonic susceptibility alone may overestimate activity in chronic or surface-associated infections.

AMP resistance is nevertheless experimentally selectable and should not be assumed to be absent [34, 49]. Envelope remodeling, peptide inactivation, efflux, and stress-response regulation can produce heritable reductions in susceptibility, whereas biofilm physiology primarily contributes phenotypic tolerance [34, 49]. Candidate evaluation should extend beyond MIC to include envelope-relevant susceptibility, serial-passage resistance testing, biofilm models, and disease-relevant environmental conditions [8, 34]. AMP efficacy is thus determined by the interaction of envelope biology, adaptive responses, and community physiology [3, 44].

**Fig. 2:**
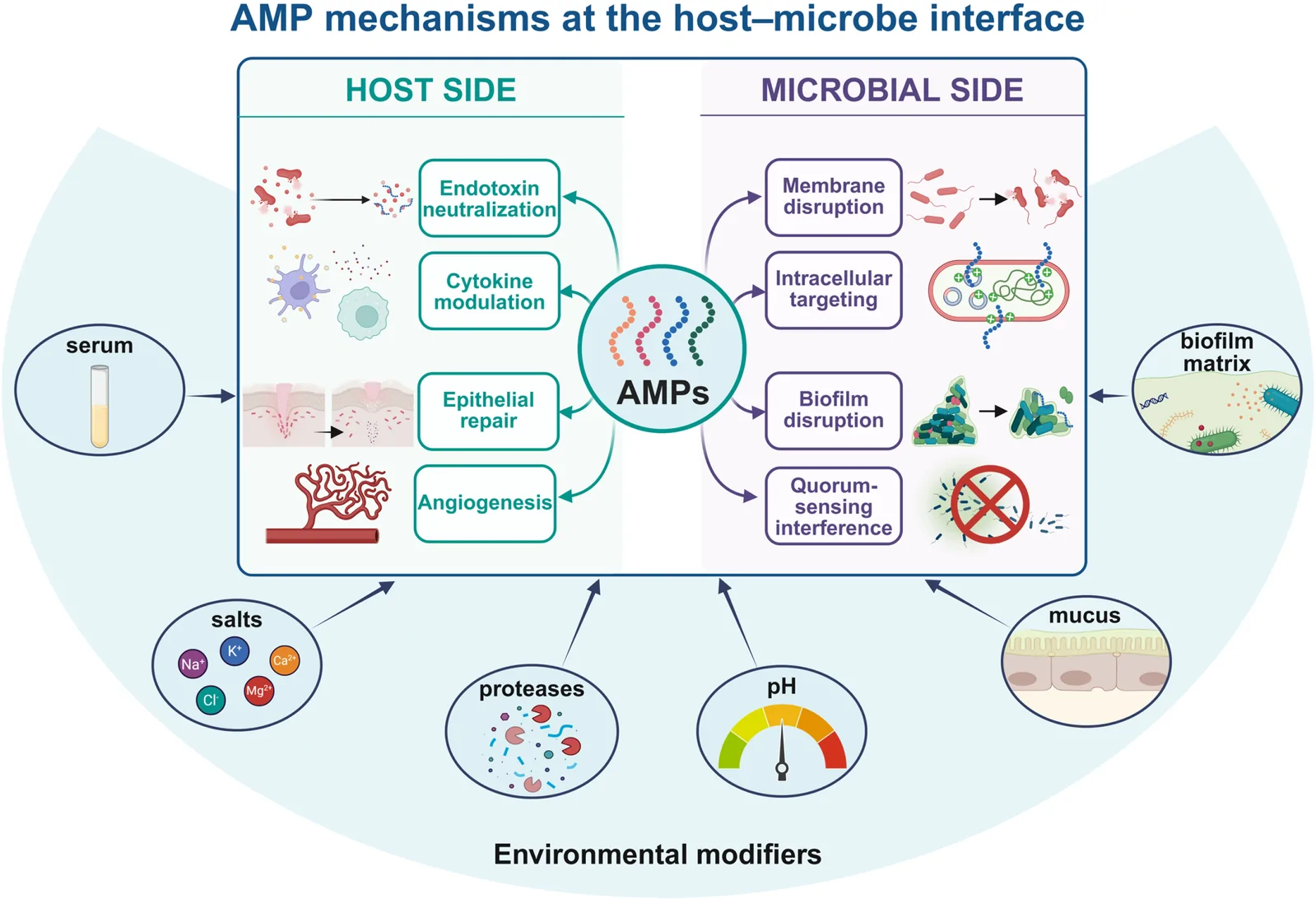
Mechanistic activities and context-dependent modifiers of antimicrobial peptides at the host–microbe interface. Antimicrobial peptides (AMPs) exert complementary activities on the microbial and host sides of the host–microbe interface. Microbe-directed activities include membrane perturbation, intracellular targeting, biofilm inhibition or disruption, and quorum-sensing interference. Host-directed activities include endotoxin neutralization, immune modulation, epithelial repair, and angiogenesis and tissue repair. The magnitude, target accessibility, and biological consequences of these activities are modified by disease-specific environmental factors, including serum proteins, salts, proteases, pH, mucus, and biofilm matrices. This figure was conceptualized by the author and created using BioRender.com.

**Mechanisms of AMPs at the host–microbe interface.** Fig. 1 presents the conceptual progression from the conventional antibiotic-substitute view to disease-context-first AMP development, whereas Fig. 2 summarizes mechanistic activities at the host–microbe interface. It distinguishes microbe-directed effects, including membrane perturbation, intracellular targeting, and biofilm inhibition or disruption, from host-directed effects such as endotoxin neutralization, immune modulation, epithelial repair, and angiogenesis. These activities are further modified by disease-specific environmental conditions.

**Direct antimicrobial mechanisms of AMPs.** Direct antibacterial mechanisms span membrane perturbation, depolarization, outer-membrane permeabilization, peptide translocation, and intracellular target engagement [3, 35, 38-42]. Many cationic AMPs first associate with negatively charged microbial surfaces and subsequently disrupt membrane organization [35, 38, 39]. In Gram-negative bacteria, interactions with LPS and lipid A can facilitate outer-membrane permeabilization and access to the cytoplasmic membrane or intracellular targets [35, 47, 48]. Membrane damage can impair ion homeostasis and energy metabolism through depolarization, leakage, or membrane-protein displacement [35, 39]. Other AMPs translocate with limited membrane disruption and inhibit nucleic-acid metabolism, translation, protein folding, cell division, or other intracellular processes [40-42]. Direct antimicrobial activity spans a mechanistic spectrum rather than a single pore-forming process.

**Antibiofilm mechanisms of AMPs.** Antibiofilm activity is mechanistically distinct from planktonic killing because biofilms combine adhesion, extracellular matrices, physiological heterogeneity, and stress-adapted cells [44, 51, 52]. AMPs can inhibit initial attachment, alter biofilm-associated signaling, penetrate or disrupt matrix-associated communities, and sensitize embedded bacteria to antimicrobial treatment [53-55]. LL-37, for example, inhibits *Pseudomonas aeruginosa* (*P. aeruginosa*) biofilm formation at subinhibitory concentrations by reducing attachment and altering biofilm-associated pathways [55]. Some engineered antibiofilm peptides also retain activity against established drug-resistant biofilms [53]. Thus, AMP efficacy against biofilms may involve both bacterial killing and modification of community-level defenses.

**Host-directed mechanisms of AMPs.** Host-defense peptides also regulate inflammation, immune-cell responses, epithelial repair, and vascular remodeling [4, 56]. LL-37 can modulate LPS- and Toll-like receptor-associated inflammatory responses rather than acting solely as an antimicrobial molecule [57-59]. Host-defense peptides can also influence chemotaxis and leukocyte functions, although these effects depend on peptide concentration, receptor usage, and tissue context [56, 57]. In barrier tissues, LL-37 contributes to epithelial migration and wound closure [60, 61], whereas LL-37/hCAP-18 promotes angiogenic responses in experimental systems [62]. These findings support consideration of selected AMPs as host-directed or tissue-supportive therapeutics in addition to direct antimicrobials.

**Context dependency of AMP mechanisms.** AMP activity is strongly influenced by the local biological environment. Ionic conditions can reduce peptide–microbe electrostatic interactions [63, 64], while serum or tissue binding may reduce the freely available peptide concentration [8]. Proteolysis can shorten peptide exposure, and mucus or extracellular DNA–actin complexes can sequester peptides and restrict their access to microbial targets [65, 66]. Local pH can further alter peptide charge and antimicrobial activity [67]. Consequently, activity measured in standardized planktonic assays may not predict efficacy in serum-rich wounds, mucus-rich airways, or biofilm-associated infections. Mechanistic evaluation should combine membrane and intracellular-target assays with antibiofilm, host-cell, and disease-relevant environmental models (Fig. 2; Supplementary Table S2) [8, 42, 54]. AMPs are thus best understood as context-dependent modulators of the host–microbe interface rather than solely as membrane-active antimicrobials.

### Disease-Context-Specific Applications of AMPs

**Preliminary efficacy of AMPs in AMR therapy.** The growing burden of AMR has increased interest in AMPs against difficult-to-treat pathogens, including the ESKAPE organisms *Enterococcus faecium*, *Staphylococcus aureus*, *Klebsiella pneumoniae*, *Acinetobacter baumannii*, *P. aeruginosa*, and *Enterobacter* spp. [68, 69]. Many candidates inhibit resistant Gram-positive and Gram-negative bacteria *in vitro*, but activity should be interpreted in relation to strain variation, bacterial physiological state, envelope architecture, biofilm formation, host fluids, and host-cell toxicity [27]. Thus, progression should depend not on MIC alone but on whether activity is retained under disease-relevant conditions and whether the resulting efficacy–safety balance is acceptable [27]. This distinction is important because peptide performance can change substantially between standardized media and host-like matrices.

A realistic near-term role for AMPs is antibiotic potentiation rather than universal replacement of conventional antibiotics [27, 70]. Membrane-active peptides can increase Gram-negative outer-membrane permeability and improve access of companion antibiotics, while selected antibiofilm peptides can enhance antibiotic activity against established biofilms [70, 71]. Accordingly, AMP–antibiotic combinations should be evaluated using checkerboard or fractional inhibitory concentration analyses and time-kill assays, together with biofilm and resistance-suppression models [70, 71]. These complementary assays distinguish simple growth inhibition from reproducible synergy, killing kinetics, and activity in structured bacterial communities. Co-administration retains formulation flexibility, whereas AMP–antibiotic conjugates seek to coordinate bacterial entry and local co-exposure [72, 73]. Combination products introduce additional challenges, including PK alignment, linker or release behavior, molecular interference, manufacturing complexity, and combined toxicity [72, 73]. These constraints favor local or adjunctive uses—including wound-directed, inhaled, and device-associated delivery—over systemic AMP monotherapy for many near-term indications (Table 1) [27].

**Applications of AMPs in diseases beyond resistant bacterial infection.** Chronic wounds are a compelling context because infection, biofilms, persistent inflammation, and impaired tissue repair coexist within the same microenvironment [4, 74]. Here, useful AMP functions extend beyond bacterial killing to antibiofilm activity, modulation of excessive inflammation, and support of epithelial repair [4, 75]. Hydrogel- or dressing-based delivery can increase local residence, limit systemic exposure, and permit combination with other antimicrobial or regenerative components [75]. Candidate testing should include biofilm-rich, protease- and exudate-containing conditions, and tissue-repair endpoints rather than standard broth susceptibility alone [74, 75].

Barrier tissues provide a second context in which local AMP delivery is biologically plausible because endogenous host-defense peptides contribute to microbial control, epithelial protection, and immune regulation [4, 76]. In oral and other mucosal settings, development should assess epithelial compatibility, local retention, mucus, protease, pH, and microbiome disturbance rather than pathogen killing alone [76]. Device-associated infections are similarly attractive because immobilized or locally released AMPs can act at biomaterial surfaces where adhesion and early biofilm formation occur [77, 78]. Translation requires durable activity after processing and sterilization, stable surface attachment or controlled release, low cytotoxicity, resistance surveillance, and preservation of implant integration [77, 78]. This indication is especially compatible with surface-localized AMP activity because therapeutic exposure can be concentrated at the site of initial colonization.

Inhaled delivery may achieve high pulmonary peptide exposure while reducing systemic exposure, making it relevant to airway infections involving resistant bacteria, mucus, and biofilms [79]. Nevertheless, airway salts, sputum components, proteases, surfactants, and mucociliary clearance can reduce peptide availability or activity [79]. Inhaled candidates require appropriate aerosol performance, epithelial safety, and retained activity in sputum-like and biofilm-associated models [79].

Some host-defense peptides also exhibit antifungal, antiviral, and immunomodulatory activities, creating potential opportunities in polymicrobial and barrier-site diseases [4, 76, 80, 81]. Such breadth should not be equated with broad clinical utility: each indication requires pathogen- and tissue-specific validation of potency, formulation, toxicity, microbiome effects, and host responses [4, 76]. Likewise, endotoxin neutralization, cytokine modulation, epithelial repair, and angiogenesis may be beneficial in selected settings but can become undesirable when host responses or repair are dysregulated [4]. The key translational question is not simply whether an AMP kills an AMR pathogen, but whether its antimicrobial, antibiofilm, delivery, and host-directed properties match the requirements of a defined disease context. In this framework, the strongest near-term role of AMPs may be potentiation and indication-specific deployment rather than universal replacement of antibiotics (Fig. 1) [27, 70].

### Engineering and Delivery of AMPs

**Engineering strategies for next-generation AMPs.** Drug-like AMP design requires simultaneous optimization of antimicrobial potency, host selectivity, stability, and manufacturability, rather than minimization of MIC alone [13, 27]. Sequence-level design remains the first layer because charge, charge distribution, hydrophobicity, amphipathicity, structural propensity, and length jointly influence bacterial membrane association and host-cell toxicity [13, 27]. Increasing cationicity can strengthen binding to anionic bacterial surfaces, whereas excessive charge may increase nonspecific interactions with host components or impair tissue penetration. Similarly, hydrophobicity and amphipathicity can improve membrane insertion but, when excessive, promote aggregation, serum binding, hemolysis, and mammalian membrane toxicity. Peptide length also creates a practical trade-off: sequences that are too short may lose structural integrity or target engagement, whereas longer peptides generally increase synthesis cost and can complicate stability and purification. Sequence optimization should balance these properties to improve therapeutic index rather than maximize membrane disruption.

Chemical modification can improve pharmacological performance when matched to the peptide mechanism. D-amino acid substitution can increase resistance to proteolysis, as demonstrated for D-Pin2 [29], whereas cyclization and hydrocarbon stapling can restrict conformation and improve stability. Importantly, a double-stapled magainin-II derivative showed increased plasma stability, low toxicity, and activity against multidrug-resistant Gram-negative bacteria in mice, providing direct evidence that structural stabilization can improve *in vivo* performance [82]. Terminal modification and lipidation can also alter charge, helicity, membrane association, and tissue retention, but excessive hydrophobic modification may increase aggregation or mammalian membrane toxicity [13, 27]. Peptidomimetics further expand the design space: peptoids and related synthetic scaffolds preserve cationic and amphiphilic pharmacophores while reducing dependence on natural α-amino acid backbones [83]. Each modification can alter target recognition, biodistribution, aggregation, tissue penetration, and toxicity. Modified candidates should be treated as new molecular entities and evaluated using antimicrobial activity, hemolysis or cytotoxicity, serum and protease stability, and disease-relevant exposure rather than MIC alone [27].

Manufacturing should also be considered during lead optimization. Solid-phase synthesis supports rapid medicinal-chemistry iteration but becomes increasingly constrained by peptide length, hydrophobic aggregation, purification yield, cost, and environmental burden at scale [13, 84]. Recombinant production can improve feasibility for selected AMPs, but host toxicity, proteolysis, folding or disulfide-pairing errors, low yield, and fusion-partner or endotoxin removal remain important limitations [84]. Thus, engineering success should be defined by a disease-specific balance of potency, safety, stability, and production feasibility.

**AI-guided discovery and delivery biotechnology of AMPs.** AI and machine learning now expand AMP discovery across peptide databases, metagenomes, proteomes, and synthetic sequence space [9, 10, 85, 86]. Explainable deep-learning and virtual-evolution approaches have generated experimentally active candidates against multidrug-resistant pathogens [9, 10], while global microbiome mining identified a large sequence space of predicted AMPs and validated selected candidates *in vitro* and in animal infection models [86]. Generative models and protein language models can further broaden this search space by proposing sequences beyond natural AMP families [85]. These approaches can prioritize sequence features, predicted activity, and selectivity more rapidly than exhaustive experimental screening, but computational novelty is not equivalent to translational suitability.

Biological validation remains the principal bottleneck because training data contain redundant sequences, heterogeneous susceptibility methods, uneven pathogen representation, and incomplete toxicity or stability annotations [85, 87]. DRAMP 4.0 partly addresses this problem by incorporating serum and protease stability, hemolysis, cytotoxicity, clinical, and patent annotations [87]. AI-derived candidates should be evaluated using external test sets, standardized antimicrobial assays, killing kinetics, host-cell toxicity, serum and protease stability, and disease-relevant biofilm or host-cell models. Closed-loop workflows that iteratively connect prediction, synthesis, experimental testing, and model updating are consequently more informative than one-pass *in silico* ranking [85].

Delivery should likewise be treated as part of AMP therapeutic design because formulation changes local exposure, stability, target access, and toxicity [27]. Nanoparticles, hydrogels, wound dressings, and surface-immobilized systems can protect peptides, increase local retention, tune release, or reduce systemic exposure. Nanoparticle-assisted AMP delivery has shown efficacy in an experimental *A. baumannii* infection model [88], illustrating how formulation can convert peptide activity into improved local exposure. Self-assembly provides a complementary strategy in which peptide molecules form nanoparticles, nanofibers, or supramolecular hydrogels with combined antimicrobial and material functions [89]. Recent primary studies show that bacteria-responsive or pH-responsive assemblies can improve local retention, proteolytic stability, biofilm control, and therapeutic efficacy in infection models [90, 91]. However, self-assembly is not intrinsically beneficial: excessive or kinetically trapped aggregation may sequester active peptide and restrict access to microbial targets. Assembly morphology, rheology, disassembly and release kinetics, cytotoxicity, manufacturability, and batch reproducibility must be optimized together with antimicrobial activity.

**Fig. 3:**
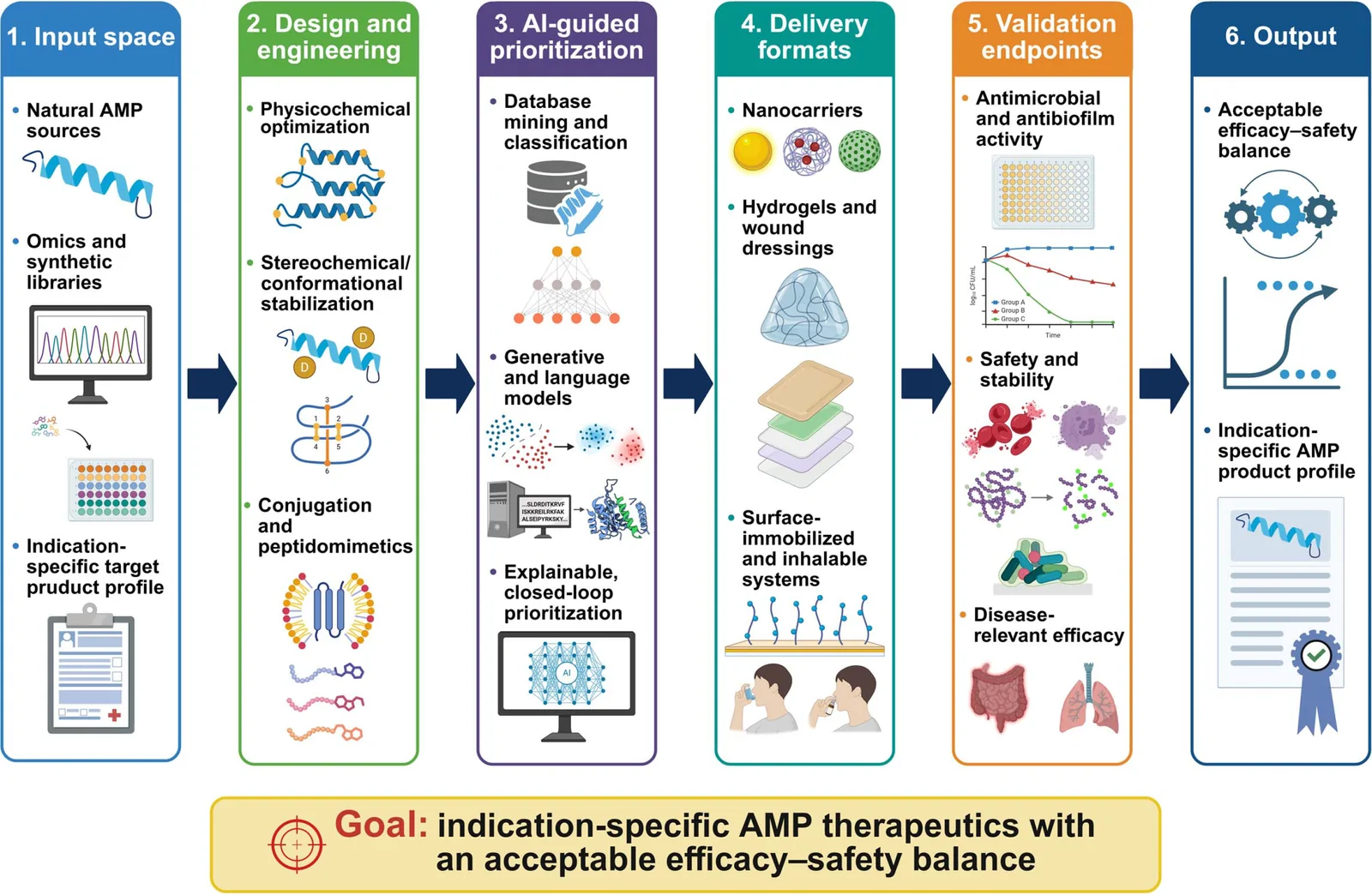
Integrated engineering, AI-guided discovery, delivery, and validation pipeline for next-generation antimicrobial peptides. This schematic illustrates an integrated development pipeline for next-generation antimicrobial peptides (AMPs). Candidate discovery begins from diverse input spaces, including natural AMPs, metagenomes or proteomes, synthetic libraries, and clinical constraints. Sequence and scaffold engineering strategies are then used to tune physicochemical and structural properties, including charge, hydrophobicity, amphipathicity, stereochemistry, conformational constraint, lipidation or PEGylation, and peptidomimetic scaffolds. Artificial intelligence (AI)-guided prioritization, including database mining, classifier-based prediction, generative modeling, protein language models, and explainable AI, expands and ranks candidate sequences but must be coupled to biological validation. Delivery formats, including nanoparticles, hydrogels, wound dressings, surface immobilization, and inhalable formulations, influence peptide exposure, stability, toxicity, and indication fit. Validation endpoints, including minimum inhibitory concentration (MIC) and minimum bactericidal concentration (MBC), time-kill kinetics, hemolysis and cytotoxicity, serum and protease stability, biofilm assays, and disease-context models, provide go/no-go criteria for translational development. The output stage depicts an acceptable efficacy–safety balance and indication-specific product profiles, rather than sequences selected by antimicrobial potency alone. This figure was conceptualized by the author and created using BioRender.com.

Overall, sequence engineering, AI-guided discovery, manufacturability, and delivery should be integrated rather than pursued as independent optimization steps. This integrated view also reduces premature lead selection based on isolated improvements that compromise other developability attributes. The appropriate endpoint is not the most potent peptide in a screening assay, but a manufacturable candidate with validated exposure, safety, and efficacy for a defined disease context (Fig. 3) [27, 85, 87].

### Translational Barriers and Developmental Framework of AMPs

**Major barriers to AMP translation.** Despite their substantial biological promise, AMPs face interdependent barriers that include resistance, host-cell toxicity, proteolytic degradation, serum binding, salt sensitivity, uncertain PK/PD behavior, manufacturing constraints, and regulatory complexity [4, 8, 34, 87]. These liabilities are mechanistically linked: physicochemical features that favor membrane association and antimicrobial potency can also increase hemolysis, cytotoxicity, aggregation, or nonspecific tissue binding, whereas proteolysis and serum binding reduce the free peptide concentration available at the infection site [4, 8, 13]. Consequently, activity measured by planktonic MIC may not predict efficacy in protein-rich, ionic, biofilm-associated, or tissue environments. Poorly defined exposure–response relationships further complicate systemic development, while sequence length, aggregation, folding, purification yield, and recombinant-host toxicity affect manufacturability [13, 84]. Clinical translation is especially sensitive to these mismatches because local residence time, release, tissue penetration, and systemic exposure jointly determine whether an effective concentration can be sustained without unacceptable toxicity. Formulation and PK/PD should be treated as integral candidate properties rather than downstream corrections. These constraints support prioritizing indications in which local or adjunctive delivery can achieve effective exposure with an acceptable safety margin [8, 27].

**Clinical lessons from failed or partially successful AMP trials.** Clinical experience demonstrates that biological activity does not necessarily translate into clinical benefit. Pexiganan, a topical magainin analog evaluated for mildly infected diabetic foot ulcers, was compared with oral ofloxacin in two randomized, double-blind trials. One trial did not demonstrate equivalence, whereas the second and pooled analyses supported comparable clinical improvement, microbiological eradication, and wound healing [92]. The program illustrates how trial design, analysis populations, patient selection, infection severity, background wound care, and equivalence criteria can affect the interpretation of an otherwise plausible local antimicrobial strategy.

Iseganan provides a stronger example of mechanism–disease mismatch. In the phase III PROMPT-CT trial for chemotherapy-associated oral mucositis, the primary endpoint was not statistically significant, although several secondary outcomes favored iseganan; interpretation was additionally complicated by a drug-dispensing error [93]. A subsequent chemotherapy trial found no significant reduction in stomatitis severity or related symptoms [94], and a radiotherapy trial in head-and-neck cancer likewise showed no reduction in ulcerative oral mucositis compared with placebo [95]. These findings suggest that predominantly antimicrobial activity is insufficient when epithelial injury and inflammatory tissue responses are major drivers of disease.

Omiganan similarly distinguishes pharmacodynamic target engagement from therapeutic efficacy. A phase II target-lesion study in atopic dermatitis identified modest local clinical and microbiological effects [96], whereas a subsequent study showed reduced *Staphylococcus* abundance and increased microbial diversity without significant improvement in clinical symptoms [97]. Collectively, these programs indicate that microbiological response should remain a secondary or mechanistic endpoint unless it is prospectively linked to clinically meaningful benefit. Negative trials should also distinguish failure of target engagement from failure of target engagement to alter disease and from inadequate formulation or exposure. Future trials should define an indication-specific target product profile, establish formulation-specific exposure–response relationships, and select patients and primary endpoints that directly reflect the proposed antimicrobial or host-directed mechanism.

**Potential unintended consequences and stewardship considerations.** AMPs should not be considered resistance-proof. Repeated exposure can select heritable reductions in susceptibility through changes in surface charge, membrane composition, envelope regulation, proteolysis, efflux, or stress responses [34, 49, 98, 99]. Experimental evolution has produced stable resistance in *S. aureus* and *Stenotrophomonas maltophilia*, including cross-resistance to endogenous host-defense peptides or conventional antibiotics [98, 99]. Importantly, resistance propensity varies among peptides; systematic experimental evolution showed that some candidates generated substantial resistance whereas others showed limited resistance under the same general framework [100]. Resistance should be treated as a candidate-specific developability liability rather than inferred from AMP class or mechanism.

Development programs should include serial-passage experiments under exposure conditions relevant to the intended formulation, confirmation of resistance stability after drug-free passage, genomic analysis of adapted isolates when appropriate, and reassessment of MIC, killing kinetics, fitness, and cross-resistance. When stable resistance emerges, fitness, virulence-associated phenotypes, susceptibility to endogenous host-defense peptides, and collateral changes in antibiotic susceptibility help determine its clinical significance. Phenotypic tolerance associated with biofilms or slow-growing populations should be distinguished from stable inherited resistance because the biological and stewardship implications differ [51, 52]. These data should contribute directly to indication-specific go/no-go decisions.[Table T3]

Therapeutic AMPs may also alter commensal communities or host responses. Intrinsic AMP resistance contributes to the persistence of selected gut commensals during inflammation, and topical omiganan modified the cutaneous microbiota without improving atopic dermatitis symptoms [97, 101]. Host-directed activities can likewise become liabilities when mismatched to tissue context. LL-37 has induced airway epithelial apoptosis under low-serum experimental conditions [102], while LL-37–self-DNA complexes activate plasmacytoid dendritic cells and type I interferon responses [103]. These findings support route- and indication-specific assessment of microbiome effects, epithelial integrity, inflammatory responses, and longitudinal safety rather than assuming that endogenous origin ensures biological safety.

**Minimum preclinical evaluation package for AMPs.** A minimum preclinical package should begin with standardized MIC testing but extend to bactericidal kinetics, biofilm activity, environmental robustness, host-cell safety, resistance, combination effects, and indication-relevant *in vivo* efficacy [104]. MBC and time-kill assays distinguish growth inhibition from killing and identify concentration dependence or regrowth. For chronic or surface-associated infections, biofilm prevention and eradication assays are necessary because biofilm susceptibility can differ markedly from planktonic susceptibility [105, 106]. Serum, physiological salt, and relevant biological matrices should be introduced early to identify protein binding or ionic loss of activity, together with protease-stability testing when degradation is likely to limit exposure [8, 63, 65, 66].

Hemolysis and cytotoxicity should be interpreted alongside antimicrobial activity to define selectivity rather than treated as late-stage safety tests. Serial passage should quantify resistance liability, and AMP–antibiotic candidates require checkerboard or fractional inhibitory concentration analyses supported by time-kill and, when relevant, biofilm-combination assays [70, 107, 108]. Finally, *in vivo* models should reproduce the intended route, formulation, and disease biology rather than merely demonstrate efficacy in an acute infection model. Additional tests should remain formulation-specific: surface-immobilized candidates require coating durability and retained activity after sterilization, whereas inhaled candidates require suitable aerosol behavior and activity in sputum-like matrices. Supplementary Table S3 can carry this assay-level detail; the main text should emphasize that advancement requires an integrated efficacy–safety–exposure assessment rather than MIC ranking alone.

**Indication-first therapeutic framework for AMPs.** An indication-first framework should begin with the unmet clinical need and limitations of current therapy, then define the AMP function, formulation, exposure profile, and evidence required for advancement. Table 1 operationalizes this sequence across resistant bacterial infection, biofilm- and device-associated infection, chronic wounds, mucosal or respiratory applications, and antibiotic-adjuvant strategies. The framework does not assume that one peptide should provide all desirable activities. Instead, each candidate–formulation combination should be matched to a specific therapeutic gap using validated antimicrobial or host-directed activity, host compatibility, disease-relevant environmental performance, and feasible exposure [8, 27].

Once this candidate–indication–formulation combination is defined, progression should depend on indication-specific go/no-go criteria that integrate resistance and cross-resistance, safety, formulation stability, local or systemic exposure, manufacturability, and efficacy in relevant biological models [8, 27, 87]. Supplementary Table S3 summarizes the general preclinical package, whereas Fig. 3 integrates discovery, engineering, AI-guided prioritization, delivery selection, and biological validation. Together, these elements convert the disease-context-first concept into a practical development framework in which clinical need determines what an AMP must do, how it should be delivered, and which evidence is required before further translation.

### Future Perspectives and Conclusion

**Disease-context-specific developmental priorities for AMPs.** Future AMP development should move from broad-spectrum potency screening toward indication-led design that aligns peptide function, formulation, exposure, safety, and validation endpoints with disease biology [4, 27]. As antimicrobial, antibiofilm, and host-directed activities are context dependent, candidate selection should include tissue-relevant conditions and, for barrier-site applications, effects on commensal communities and epithelial compatibility [4, 56, 76]. AI and machine learning can broaden sequence discovery [85, 86], but computational prioritization should remain coupled to experimental assessment of antimicrobial activity, toxicity, stability, and performance in disease-relevant models [85, 87].

Near-term translational priorities should emphasize indications in which AMPs provide functions that conventional antibiotics do not adequately deliver. Antibiotic potentiation remains attractive, particularly where membrane permeabilization or biofilm disruption improves companion-drug activity [70, 71]. Localized or inhaled formulations may improve site-specific exposure and reduce systemic liability, but must be evaluated for release behavior, tissue compatibility, stability, and manufacturability [27, 79]. Host-directed functions may also be valuable, but only when they improve disease-relevant outcomes without unacceptable inflammatory or tissue effects [4, 56].

**Concluding remarks: AMPs as multifunctional therapeutic platforms.** AMPs should therefore be developed not as universal antibiotic replacements but as multifunctional therapeutic platforms at the host–microbe interface. The central criterion is an indication-specific balance of potency, selectivity, stability, manufacturability, delivery feasibility, and biological validation [4, 27, 87]. A translational framework integrating mechanism, microbial susceptibility, host response, formulation, safety, resistance risk, and disease-relevant efficacy provides a more realistic basis for candidate advancement than MIC ranking alone (Table 1; Fig. 3). This framework preserves the therapeutic potential of AMPs while acknowledging their biological and pharmacological constraints and supports deployment only when peptide function and delivery are matched to a defined disease context (Fig. 1) [4, 56].

## Supplemental Materials

Supplementary data for this paper are available on-line only at http://jmb.or.kr.



## Figures and Tables

**Table 1:** Therapeutic-context framework for AMP development: clinical challenges, treatment gaps, complementary functions, and formulation strategies.

Therapeutic context or strategy	Major clinical or therapeutic challenge	Limitations of current treatment	Potential AMP advantages or complementary functions^a^	Potential applications or formulation strategies	References
MDR bacterial infection	Drug resistance; Gram-negative envelope barriers; rapid progression	Few active agents; toxicity of some last-line drugs; poor target access	Rapid killing; membrane permeabilization; activity against selected resistant isolates; antibiotic potentiation	Local therapy; AMP–antibiotic combination or co-delivery; systemic use after adequate PK/PD and safety evaluation	[1, 3, 8, 70, 71, 108]
Biofilm- and device-associated infection	Adhesion; matrix formation; persister-like physiology; recurrent colonization	Poor antibiotic penetration; phenotypic tolerance; recurrence; device removal	Anti-adhesion; biofilm inhibition or disruption; sensitization of biofilm cells; surface-localized activity	Immobilized or controlled-release coating; hydrogels; peri-device depots	[51, 109-111]
Chronic wound	Biofilm burden; chronic inflammation; impaired re-epithelialization and closure	Incomplete biofilm control; persistent inflammation; variable healing	Antimicrobial and antibiofilm activity; inflammation modulation; epithelial migration; angiogenesis and repair	Topical gel or cream; AMP-loaded hydrogel; wound dressing; local depot	[6, 74, 75, 112, 113]
Mucosal or respiratory infection and barrier disorder	Mucus or sputum; proteases; altered ionic conditions; rapid clearance	Inadequate local exposure; systemic toxicity; reduced activity in airway or mucosal matrices	Local antimicrobial and antibiofilm activity; barrier support; context-dependent immune modulation	Inhaled aerosol or dry powder; mucosal carrier; nanoparticles; mucoadhesive or mucus-penetrating system	[76, 79, 114]
Antibiotic-adjuvant therapy	Restricted antibiotic access; envelope barriers; biofilm or persister tolerance	Dose escalation may increase toxicity; poor penetration limits monotherapy	Membrane permeabilization; increased antibiotic uptake; biofilm sensitization; restoration of antibiotic activity	Co-administration; fixed combination; AMP–antibiotic conjugate; co-loaded nanocarrier	[8, 70, 71, 108, 115]

^a^Potential advantages are candidate- and context-dependent and should not be interpreted as universal properties of all AMPs. Each proposed function requires experimental confirmation under conditions relevant to the intended indication and formulation.

General developability and preclinical evaluation requirements, including antimicrobial activity, resistance and cross-resistance, host-cell safety, serum and protease stability, environmental tolerance, formulation performance, and disease-relevant validation, are summarized in Supplementary Table S3.

Abbreviations: AMP, antimicrobial peptide; MDR, multidrug-resistant; PK/PD, pharmacokinetics/pharmacodynamics.

**Table 2:** Microbiological determinants of antimicrobial peptide susceptibility and tolerance.

Determinant	Microbial feature	Effect on AMP susceptibility	Suggested assay/readout	References
Gram-positive envelope	Peptidoglycan; wall and lipoteichoic acids	Alters peptide binding, trapping, diffusion, and membrane access	Species-panel MIC; cell-wall mutant comparison	[45, 46]
Gram-negative envelope	Outer membrane; LPS; lipid A; capsule	Restricts binding, uptake, and inner-membrane access	LPS/lipid A mutants; outer-membrane permeability assay	[47, 48]
Surface-charge modification	D-alanylation; MprF; lipid A modification	Reduces electrostatic AMP binding	Mutant/complementation assay; zeta potential	[34, 46, 47]
Proteolysis and efflux	Secreted proteases; peptide efflux systems	Degrades or removes active peptide	Protease-stability assay; efflux mutant comparison	[34, 43, 49]
Envelope-stress response	Two-component systems; membrane remodeling	Promotes adaptive tolerance or resistance	Stress-gene expression; resistance passage	[43, 47]
Acquired resistance and cross-resistance	Stable mutations or regulatory adaptation after AMP exposure	Reduced AMP susceptibility; possible cross-resistance to other peptides or antibiotics	Serial passage; stability testing; WGS; cross-resistance profiling	[98-100, 116-119]
Biofilm matrix	EPS; eDNA; proteins; polysaccharides	Limits diffusion and sequesters cationic AMPs	Biofilm inhibition/eradication; penetration imaging	[44, 52]
Persister/community physiology	Slow growth; nutrient limitation; oxygen gradients	Phenotypic tolerance despite planktonic susceptibility	Time-kill; persister assay; biofilm viability	[51, 120]

Abbreviations: AMP, antimicrobial peptide; eDNA, extracellular DNA; EPS, extracellular polymeric substances; LPS, lipopolysaccharide; MIC, minimum inhibitory concentration; WGS, whole-genome sequencing.

**Table 3 T3:** Representative clinically evaluated antimicrobial peptides and AMP-derived candidates: mechanisms, clinical development outcomes, and translational limitations.

Candidate and parent scaffold or molecular origin	Principal mechanism(s)^a^	Clinical context and formulation	Clinical development and reported outcome	Key limitations or translational lessons^b^	References
Pexiganan (MSI-78); magainin-2 analog	Amphipathic membrane insertion, membrane permeabilization, pore formation, and membrane fragmentation	Topical cream for mildly infected diabetic foot ulcers	Inconsistent equivalence results across two pivotal trials; one trial and pooled analysis supported outcomes comparable to oral ofloxacin.	Trial-to-trial inconsistency and sensitivity to study design and wound-care context; experimentally selectable resistance with reported cross-resistance.	[92, 118, 119, 121]
Iseganan (IB-367); protegrin-1 analog	Rapid membrane permeabilization and broad microbicidal activity	Oral rinse for chemotherapy- or radiotherapy-associated oral mucositis	Favorable secondary signals in the initial phase III study; no significant clinical benefit in subsequent phase III trials.	Limited alignment with multifactorial mucositis pathobiology; possible vehicle or oral-care effects and no reproducible primary-endpoint benefit.	[93-95]
Omiganan (MBI-226); indolicidin analog	Membrane-active antimicrobial activity and modulation of local staphylococcal burden	Topical gel for mild-to-moderate atopic dermatitis	Reduced *Staphylococcus* abundance and altered microbial diversity; no significant improvement in clinical symptoms in the larger phase II study.	Microbiological target engagement did not translate into clinical efficacy; microbiome modification alone was insufficient.	[96, 97]
LL-37 (ropocamptide); human cathelicidin	Antimicrobial activity, immunomodulation, epithelial migration, angiogenesis, and wound repair	Topical treatment combined with compression therapy for hard-to-heal venous leg ulcers	Improved healing parameters in an initial small trial; no overall phase IIb benefit, with a post hoc signal in patients with larger ulcers.	Nonlinear dose response and heterogeneous wound biology; the subgroup signal requires prospective validation	[112, 113]
LTX-109; AMP-inspired membrane-active peptidomimetic	Binding to negatively charged bacterial membrane components, followed by rapid membrane disruption and cell lysis	Topical intranasal hydrogel for persistent MRSA or MSSA carriage	Rapid dose-dependent reduction in nasal bacterial burden during treatment; durability of decolonization remained limited or uncertain during follow-up.	Small exploratory study, transient decolonization, and local tolerability concerns.	[122]
P60.4Ac/OP-145; LL-37-derived synthetic peptide	Antibacterial and antibiofilm activity, membrane permeabilization, lipopolysaccharide neutralization, and anti-inflammatory activity	Ototopical drops for therapy-resistant chronic suppurative otitis media	Randomized phase IIa treatment success of 47% versus 6% with placebo following dose-ranging evaluation.	Small, short-duration, indication-specific trial; larger confirmatory studies are required.	[123]
hLF1-11; N-terminal lactoferrin-derived peptide	Binding to anionic microbial components, microbial-envelope disruption, direct and phagocyte-mediated antimicrobial activity, and immunomodulation	Intravenous administration evaluated in healthy volunteers and autologous HSCT recipients	Single or repeated doses up to 5 mg were generally tolerated; the studies primarily assessed safety rather than efficacy.	Limited patient exposure and no established clinical efficacy; reversible transaminase elevations require monitoring.	[124]

^a^Principal mechanisms are simplified to emphasize the clinically relevant dominant activities of each candidate and do not imply that any candidate acts through a single exclusive mechanism.

^b^The listed limitations represent candidate- and study-specific translational lessons and should not be interpreted as universal limitations of the corresponding AMP class. The candidates are representative rather than exhaustive, and the reported outcomes reflect the cited peer-reviewed studies without indicating current regulatory approval status.

Abbreviations: AMP, antimicrobial peptide; MRSA, methicillin-resistant *Staphylococcus aureus*; MSSA, methicillin-sensitive *Staphylococcus aureus*; HSCT, hematopoietic stem-cell transplantation.
