# Supplementary material for: Reframing Antimicrobial Peptides beyond Direct Antimicrobial Activity toward Host-Directed Functions and Disease-Specific Therapeutic Applications
Source: J Microbiol Biotechnol. 2026 Sep 2;36:e2606034. doi: 10.4014/jmb.2606.06034 (PMC13555005; doi:10.4014/jmb.2606.06034)
Supplement: Supplementary file 1 [file jmb-36-e2606034-supple.pdf]

J. Microbiol. Biotechnol. 2026. 36: e2606034

https://doi.org/10.4014/jmb.2606.06034

Supplementary Tables

Reframing Antimicrobial Peptides Beyond Direct Antimicrobial Activity Toward Host-Directed Functions and Disease-Specific Therapeutic Applications

Minho Lee[1,2*]

[1]*Department of Microbiology, College of Medicine, Hallym University, Chuncheon 24252, Republic of Korea*

[2]*Institute of Medical Science, College of Medicine, Hallym University, Chuncheon 24252, Republic of Korea*

[*]Corresponding author

Minho Lee, E-mail: mlee@hallym.ac.kr

Table S1. Representative natural AMP families and engineered scaffolds relevant to therapeutic development.

Biological source, AMP family, or engineered scaffold	Representative examples[a]	Characteristic structural features[b]	Notable biological activities or developmental strengths[b]	Principal developmental limitations[b]	References
Vertebrate defensins	Human β-defensin 3; α-/β-defensins; θ-defensins	Cysteine-rich; disulfide-stabilized; mainly β-sheet	Broad antimicrobial activity; epithelial defense; immune recruitment	Complex folding; salt/context dependence; delivery constraints	[1-4]
Vertebrate cathelicidins	LL-37; CRAMP; protegrins	Cationic; often amphipathic; structure induced by membrane environments	Antimicrobial activity; endotoxin neutralization; immunomodulation; epithelial repair	Proteolysis; concentration-dependent cytotoxicity; context-dependent inflammation	[1, 2, 5, 6]

Fungal defensins	Plectasin	Cysteine-stabilized defensin-like fold	Potent activity against selected Gram-positive pathogens; *in vivo* efficacy	Restricted spectrum; production and delivery requirements	[7]
Bacterial bacteriocins	Nisin; pediocin-like bacteriocins; microcins	Ribosomal; often post-translationally modified	Targeted antibacterial activity; lipid II-dependent action in nisin	Narrow spectrum; resistance; formulation and regulatory constraints	[8-10]
Insect AMPs	Cecropin A; insect defensins; attacins; drosomycin	α-Helical, cysteine-rich, glycine-rich, or proline-rich	Rapid antimicrobial activity; diverse engineering scaffolds	Proteolysis; variable mammalian-cell selectivity	[11-14]
Marine AMPs	Piscidins; arenicins; tachyplesins; mytilins	α-Helical, β-hairpin, or cysteine-stabilized	Potent membrane-associated activity; structural diversity	Cytotoxicity/hemolysis in some peptides; matrix dependence	[11, 15, 16]
Plant AMPs	Defensins; thionins; snakins; cycloviolacin	Often cysteine-rich; some cyclic backbones	Antifungal/antibacterial activity; scaffold	Sequence-dependent activity; toxicity;	[11, 17, 18]

	O2		stability	production and delivery barriers	
Cyclic peptides	Cyclotides; θ-defensin analogues	Conformationally constrained; backbone or side-chain cyclization	Protease resistance; enhanced conformational stability	Synthetic complexity; altered membrane interactions; toxicity risk	[11, 12, 19]
D-amino acid-containing peptides	D-enantiomers; retro-inverso AMPs	Partial or complete L-to-D substitution	Protease resistance; metabolic stability	Activity retention and toxicity require revalidation	[12, 20]
Stapled peptides	Hydrocarbon-stapled α-helical AMPs	Conformationally stabilized α-helix	Enhanced helicity and protease resistance; tunable membrane interaction	Synthetic complexity; hydrophobicity-dependent toxicity	[11, 12, 21]
Lipopeptides	Daptomycin-like; polymyxin-like; lipidated AMPs	Peptide linked to lipid or acyl chain	Strong membrane association; potent activity; adjuvant	Aggregation; serum binding; class-dependent toxicity	[22-24]

			potential		
Peptidomimetics and synthetic mimics	Peptoids; β-peptides; arylamides; polymeric mimics	Non-natural backbone or amphiphilic scaffold	Protease stability; physicochemical tunability	Selectivity and toxicity validation; regulatory uncertainty	[11, 12, 25]

[a]Representative examples are not exhaustive.

[b]Structural features, biological activities, developmental strengths, and limitations are family- or scaffold-level summaries and may differ substantially among individual sequences, formulations, target organisms, and disease contexts.

Abbreviations: AMP, antimicrobial peptide; CRAMP, cathelicidin-related antimicrobial peptide.

Table S2. Mechanistic assays for antimicrobial peptide evaluation.

Mechanistic domain	Mechanistic activity or process	Suggested assay	Key readout	Major limitation	References
Membrane disruption	Permeabilization; bilayer destabilization; leakage	Liposome leakage; PI/SYTOX uptake; electron microscopy	Leakage; membrane damage; morphology	Model membranes do not reproduce full bacterial-envelope complexity	[22, 23, 26]
Membrane depolarization	Loss of membrane potential	$DiSC_3(5)$ or related potential-sensitive dyes	Fluorescence shift	Depolarization may be secondary to another lethal event	[22, 23, 26]
Outer-membrane permeabilization	LPS interaction; self-promoted uptake	NPN uptake; LPS-binding assay; envelope mutants	Increased permeability or altered mutant susceptibility	Does not establish inner-membrane killing	[26-28]

Intracellular targeting	Inhibition of nucleic acids, translation, folding, division, or enzymes	Peptide uptake; biosynthesis assay; target-specific assay	Intracellular localization or target inhibition	Difficult to distinguish from secondary effects of membrane damage	[26, 29, 30]
Biofilm inhibition or disruption	Adhesion inhibition; matrix disruption; biofilm killing	Crystal violet, MBEC; CFU; confocal microscopy	Biomass, viability, penetration, or matrix disruption	Biomass loss does not necessarily indicate killing	[31-33]
Quorum-sensing interference	Altered autoinducer signaling or virulence regulation	Reporter assay; autoinducer quantification; qPCR	Signal or target-gene reduction	Reduced signaling may reflect growth inhibition	[32-34]
Endotoxin neutralization	LPS binding; suppression of LPS-induced signaling	LAL; LPS binding; TLR4/NF-κB reporter; cytokines	Reduced LPS or inflammatory activity	*In vitro* neutralization may not predict *in vivo* inflammation	[35-37]
Immune modulation	Cytokine/chemokine modulation; immune-cell recruitment	ELISA/multiplex; qPCR; migration assay	Cytokine profile or migration	Strongly peptide-, dose-, cell-, and stimulus-dependent	[2, 35, 37]

Epithelial repair	Migration; re-epithelialization; barrier recovery	Scratch assay; TEER; proliferation assay	Migration, closure, or barrier integrity	Cell-line effects may not reproduce primary tissue	[38, 39]
Angiogenesis	Endothelial migration; neovascularization	Tube formation; migration; CAM or ischemia model	Tube formation or perfusion recovery	Context- and dose-dependent; may not always be desirable	[1, 40]
Environmental tolerance	Activity in serum, salts, proteases, pH, mucus, or matrix	Modified MIC/time-kill; binding/stability; diffusion assay	Retained activity, stability, free peptide, penetration	Assay conditions lack standardization	[41-43]

Abbreviations: AMP, antimicrobial peptide; CAM, chorioallantoic membrane; CFU, colony-forming unit; $DiSC_3(5)$, 3,3'-dipropylthiadicarbocyanine iodide; ELISA, enzyme-linked immunosorbent assay; LAL, *Limulus* amebocyte lysate; LPS, lipopolysaccharide; MBEC, minimum biofilm eradication concentration; MIC, minimum inhibitory concentration; NF-κB, nuclear factor kappa-light-chain-enhancer of activated B cells; NPN, N-phenyl-1-naphthylamine; PI, propidium iodide; qPCR, quantitative polymerase chain reaction; TEER, transepithelial electrical resistance; TLR4, Toll-like receptor 4.

Table S3. Minimum preclinical evaluation package for antimicrobial peptide candidates.

Evaluation domain	Primary purpose	Core assays/readouts	Decision value	Most relevant developmental contexts	References
MIC and MBC	Establish baseline inhibitory and bactericidal activity	Broth/agar MIC; MBC	Defines initial activity window but not efficacy–safety balance	All candidates; early screening	[44-46]
Time-kill kinetics	Determine rate and extent of killing	CFU over time; concentration-response	Distinguishes rapid killing, delayed killing, regrowth, or stasis	Bactericidal candidates	[45, 46]
Biofilm prevention/eradication	Test activity against surface- and matrix-associated cells	Inhibition assay; MBEC; CFU; confocal imaging	Establishes activity beyond planktonic MIC	Wounds; device; respiratory biofilms	[47, 48]
Serum and salt tolerance	Test activity under physiologically	Modified MIC/time-kill; serum-binding	Identifies ionic shielding or protein-	Pulmonary, mucosal, wound, systemic	[41, 49, 50]

	relevant ionic/protein conditions		binding losses	applications	
Protease stability	Assess susceptibility to degradation	Protease incubation; LC-MS/HPLC; retained activity	Defines exposure durability and stabilization need	Wounds, mucosa, respiratory tract, systemic use	[41, 50, 51]
Hemolysis and cytotoxicity	Define host-cell safety and selectivity	RBC hemolysis; relevant mammalian-cell assays	Determines separation of antimicrobial activity from host toxicity	All candidates	[12, 41, 50]
Resistance serial passage	Measure resistance and cross-resistance risk	Repeated exposure; post-passage MIC; cross-resistance panel	Detects stable susceptibility shifts	Repeated-use and long-exposure applications	[12, 52, 53]
Antibiotic synergy	Evaluate adjuvant potential	Checkerboard/FICI; time-kill synergy; biofilm combination	Determines enhancement beyond additivity	Antibiotic-adjuvant and MDR/biofilm applications	[54-56]
Disease-relevant *in*	Test efficacy and safety	Wound, device,	Integrates tissue,	Lead-stage indication-	[1, 12, 41]

vivo model	in indication-matched biology	pulmonary, mucosal, or combination model	immune, PK/PD, and delivery constraints	specific development

Note: This table represents a minimum decision framework. Additional assays should be selected according to peptide mechanism, formulation, target organism, route of administration, and intended indication.

Abbreviations: AMP, antimicrobial peptide; CFU, colony-forming unit; FICI, fractional inhibitory concentration index; HPLC, high-performance liquid chromatography; LC-MS, liquid chromatography-mass spectrometry; MBC, minimum bactericidal concentration; MBEC, minimum biofilm eradication concentration; MDR, multidrug-resistant; MIC, minimum inhibitory concentration; PK/PD, pharmacokinetics/pharmacodynamics; RBC, red blood cell.

References

1. Mookherjee N, Anderson MA, Haagsman HP, Davidson DJ. 2020. Antimicrobial host defence peptides: functions and clinical potential. *Nat. Rev. Drug Discov.* **19:** 311–332. https://doi.org/10.1038/s41573-019-0058-8.
2. Mansour SC, Pena OM, Hancock RE. 2014. Host defense peptides: front-line immunomodulators. *Trends Immunol.* **35:** 443–450. https://doi.org/10.1016/j.it.2014.07.004.
3. Ganz T. 2003. Defensins: antimicrobial peptides of innate immunity. *Nat. Rev. Immunol.* **3:** 710–720. https://doi.org/10.1038/nri1180.
4. Garcia JR, Jaumann F, Schulz S, Krause A, Rodriguez-Jimenez J, Forssmann U*, et al.* 2001. Identification of a novel, multifunctional beta-defensin (human beta-defensin 3) with specific antimicrobial activity. Its interaction with plasma membranes of *Xenopus* oocytes and the induction of macrophage chemoattraction. *Cell Tissue Res.* **306:** 257–264. https://doi.org/10.1007/s004410100433.
5. Hancock RE, Sahl HG. 2006. Antimicrobial and host-defense peptides as new anti-infective therapeutic strategies. *Nat. Biotechnol.* **24:** 1551–1557. https://doi.org/10.1038/nbt1267.
6. Tokumaru S, Sayama K, Shirakata Y, Komatsuzawa H, Ouhara K, Hanakawa Y*, et al.* 2005. Induction of keratinocyte migration via transactivation of the epidermal growth factor receptor by the antimicrobial peptide LL-37. *J. Immunol.* **175:** 4662–4668. https://doi.org/10.4049/jimmunol.175.7.4662.
7. Mygind PH, Fischer RL, Schnorr KM, Hansen MT, Sonksen CP, Ludvigsen S*, et al.* 2005. Plectasin is a peptide antibiotic with therapeutic potential from a saprophytic fungus. *Nature.* **437:** 975–980. https://doi.org/10.1038/nature04051.
8. Cotter PD, Ross RP, Hill C. 2013. Bacteriocins - a viable alternative to antibiotics? *Nat. Rev. Microbiol.* **11:** 95–105. https://doi.org/10.1038/nrmicro2937.
9. Soltani S, Hammami R, Cotter PD, Rebuffat S, Said LB, Gaudreau H*, et al.* 2021. Bacteriocins as a new generation of antimicrobials: toxicity aspects and regulations. *FEMS Microbiol. Rev.* **45:** fuaa039. https://doi.org/10.1093/femsre/fuaa039.
10. Wiedemann I, Benz R, Sahl HG. 2004. Lipid II-mediated pore formation by the peptide antibiotic nisin: a black lipid membrane study. *J. Bacteriol.* **186:** 3259–3261. https://doi.org/10.1128/JB.186.10.3259-3261.2004.
11. Gan BH, Gaynord J, Rowe SM, Deingruber T, Spring DR. 2021. The multifaceted nature of antimicrobial peptides: current synthetic chemistry approaches and future directions. *Chem. Soc. Rev.* **50:** 7820–7880. https://doi.org/10.1039/d0cs00729c.
12. Magana M, Pushpanathan M, Santos AL, Leanse L, Fernandez M, Ioannidis A*, et al.* 2020. The value of antimicrobial peptides in the age of resistance. *Lancet Infect. Dis.* **20:** e216–e230. https://doi.org/10.1016/S1473-3099(20)30327-3.
13. Bulet P, Stocklin R. 2005. Insect antimicrobial peptides: structures, properties and gene regulation. *Protein Pept. Lett.* **12:** 3–11. https://doi.org/10.2174/0929866053406011.
14. Silvestro L, Weiser JN, Axelsen PH. 2000. Antibacterial and antimembrane activities of cecropin A in *Escherichia coli*. *Antimicrob. Agents Chemother.* **44:** 602–607. https://doi.org/10.1128/AAC.44.3.602-607.2000.

15. Wang S, Fan L, Pan H, Li Y, Qiu Y, Lu Y. 2023. Antimicrobial peptides from marine animals: Sources, structures, mechanisms and the potential for drug development. *Front. Mar. Sci.* **9:** 1112595. https://doi.org/10.3389/fmars.2022.1112595.
16. Campagna S, Saint N, Molle G, Aumelas A. 2007. Structure and mechanism of action of the antimicrobial peptide piscidin. *Biochemistry.* **46:** 1771–1778. https://doi.org/10.1021/bi0620297.
17. Nawrot R, Barylski J, Nowicki G, Broniarczyk J, Buchwald W, Gozdzicka-Jozefiak A. 2014. Plant antimicrobial peptides. *Folia Microbiol. (Praha).* **59:** 181–196. https://doi.org/10.1007/s12223-013-0280-4.
18. Pranting M, Loov C, Burman R, Goransson U, Andersson DI. 2010. The cyclotide cycloviolacin O2 from *Viola odorata* has potent bactericidal activity against Gram-negative bacteria. *J. Antimicrob. Chemother.* **65:** 1964–1971. https://doi.org/10.1093/jac/dkq220.
19. Craik DJ, Du J. 2017. Cyclotides as drug design scaffolds. *Curr. Opin. Chem. Biol.* **38:** 8–16. https://doi.org/10.1016/j.cbpa.2017.01.018.
20. Carmona G, Rodriguez A, Juarez D, Corzo G, Villegas E. 2013. Improved protease stability of the antimicrobial peptide Pin2 substituted with D-amino acids. *Protein J.* **32:** 456–466. https://doi.org/10.1007/s10930-013-9505-2.
21. You Y, Liu H, Zhu Y, Zheng H. 2023. Rational design of stapled antimicrobial peptides. *Amino Acids.* **55:** 421–442. https://doi.org/10.1007/s00726-023-03245-w.
22. Nguyen LT, Haney EF, Vogel HJ. 2011. The expanding scope of antimicrobial peptide structures and their modes of action. *Trends Biotechnol.* **29:** 464–472. https://doi.org/10.1016/j.tibtech.2011.05.001.
23. Hollmann A, Martinez M, Maturana P, Semorile LC, Maffia PC. 2018. Antimicrobial Peptides: Interaction With Model and Biological Membranes and Synergism With Chemical Antibiotics. *Front. Chem.* **6:** 204. https://doi.org/10.3389/fchem.2018.00204.
24. Cochrane SA, Vederas JC. 2016. Lipopeptides from *Bacillus* and *Paenibacillus* spp.: A Gold Mine of Antibiotic Candidates. *Med. Res. Rev.* **36:** 4–31. https://doi.org/10.1002/med.21321.
25. Chongsiriwatana NP, Barron AE. 2010. Comparing bacterial membrane interactions of antimicrobial peptides and their mimics. *Methods Mol. Biol.* **618:** 171–182. https://doi.org/10.1007/978-1-60761-594-1_12.
26. Brogden KA. 2005. Antimicrobial peptides: pore formers or metabolic inhibitors in bacteria? *Nat. Rev. Microbiol.* **3:** 238–250. https://doi.org/10.1038/nrmicro1098.
27. Nikaido H. 2003. Molecular basis of bacterial outer membrane permeability revisited. *Microbiol. Mol. Biol. Rev.* **67:** 593–656. https://doi.org/10.1128/MMBR.67.4.593-656.2003.
28. Raetz CR, Reynolds CM, Trent MS, Bishop RE. 2007. Lipid A modification systems in gram-negative bacteria. *Annu. Rev. Biochem.* **76:** 295–329. https://doi.org/10.1146/annurev.biochem.76.010307.145803.
29. Cardoso MH, Meneguetti BT, Costa BO, Buccini DF, Oshiro KGN, Preza SLE*, et al.* 2019. Non-Lytic Antibacterial Peptides That Translocate Through Bacterial Membranes to Act on Intracellular Targets. *Int. J. Mol. Sci.* **20:** 4877. https://doi.org/10.3390/ijms20194877.

30. Le CF, Fang CM, Sekaran SD. 2017. Intracellular Targeting Mechanisms by Antimicrobial Peptides. *Antimicrob. Agents Chemother.* **61:** e02340-16. https://doi.org/10.1128/AAC.02340-16.
31. de la Fuente-Nunez C, Reffuveille F, Mansour SC, Reckseidler-Zenteno SL, Hernandez D, Brackman G*, et al.* 2015. D-enantiomeric peptides that eradicate wild-type and multidrug-resistant biofilms and protect against lethal *Pseudomonas aeruginosa* infections. *Chem. Biol.* **22:** 196–205. https://doi.org/10.1016/j.chembiol.2015.01.002.
32. Jorge P, Lourenco A, Pereira MO. 2012. New trends in peptide-based anti-biofilm strategies: a review of recent achievements and bioinformatic approaches. *Biofouling.* **28:** 1033–1061. https://doi.org/10.1080/08927014.2012.728210.
33. Pletzer D, Hancock RE. 2016. Antibiofilm Peptides: Potential as Broad-Spectrum Agents. *J. Bacteriol.* **198:** 2572–2578. https://doi.org/10.1128/JB.00017-16.
34. Overhage J, Campisano A, Bains M, Torfs EC, Rehm BH, Hancock RE. 2008. Human host defense peptide LL-37 prevents bacterial biofilm formation. *Infect. Immun.* **76:** 4176–4182. https://doi.org/10.1128/IAI.00318-08.
35. Scott MG, Davidson DJ, Gold MR, Bowdish D, Hancock RE. 2002. The human antimicrobial peptide LL-37 is a multifunctional modulator of innate immune responses. *J. Immunol.* **169:** 3883–3891. https://doi.org/10.4049/jimmunol.169.7.3883.
36. Rosenfeld Y, Papo N, Shai Y. 2006. Endotoxin (lipopolysaccharide) neutralization by innate immunity host-defense peptides. Peptide properties and plausible modes of action. *J. Biol. Chem.* **281:** 1636–1643. https://doi.org/10.1074/jbc.M504327200.
37. Mookherjee N, Brown KL, Bowdish DM, Doria S, Falsafi R, Hokamp K*, et al.* 2006. Modulation of the TLR-mediated inflammatory response by the endogenous human host defense peptide LL-37. *J. Immunol.* **176:** 2455–2464. https://doi.org/10.4049/jimmunol.176.4.2455.
38. Heilborn JD, Nilsson MF, Kratz G, Weber G, Sorensen O, Borregaard N*, et al.* 2003. The cathelicidin anti-microbial peptide LL-37 is involved in re-epithelialization of human skin wounds and is lacking in chronic ulcer epithelium. *J. Invest. Dermatol.* **120:** 379–389. https://doi.org/10.1046/j.1523-1747.2003.12069.x.
39. Shaykhiev R, Beisswenger C, Kandler K, Senske J, Puchner A, Damm T*, et al.* 2005. Human endogenous antibiotic LL-37 stimulates airway epithelial cell proliferation and wound closure. *Am. J. Physiol. Lung Cell. Mol. Physiol.* **289:** L842–L848. https://doi.org/10.1152/ajplung.00286.2004.
40. Koczulla R, von Degenfeld G, Kupatt C, Krotz F, Zahler S, Gloe T*, et al.* 2003. An angiogenic role for the human peptide antibiotic LL-37/hCAP-18. *J. Clin. Invest.* **111:** 1665–1672. https://doi.org/10.1172/JCI17545.
41. Dad N, Elsawy MA, Humphreys G, Pluen A, Lu JR, McBain AJ. 2025. A critical view of antimicrobial peptides: exploring their potential and the barriers to realization. *J. Appl. Microbiol.* **136:** lxaf087. https://doi.org/10.1093/jambio/lxaf087.
42. Bucki R, Byfield FJ, Janmey PA. 2007. Release of the antimicrobial peptide LL-37 from DNA/F-actin bundles in cystic fibrosis sputum. *Eur. Respir. J.* **29:** 624–632. https://doi.org/10.1183/09031936.00080806.
43. Malik E, Dennison SR, Harris F, Phoenix DA. 2016. pH Dependent Antimicrobial Peptides and Proteins, Their Mechanisms of Action and Potential as Therapeutic Agents.

Pharmaceuticals (Basel). **9:** 67. https://doi.org/10.3390/ph9040067.
44. Wiegand I, Hilpert K, Hancock RE. 2008. Agar and broth dilution methods to determine the minimal inhibitory concentration (MIC) of antimicrobial substances. *Nat. Protoc.* **3:** 163–175. https://doi.org/10.1038/nprot.2007.521.
45. Balouiri M, Sadiki M, Ibnsouda SK. 2016. Methods for *in vitro* evaluating antimicrobial activity: A review. *J. Pharm. Anal.* **6:** 71–79. https://doi.org/10.1016/j.jpha.2015.11.005.
46. Barry AL. 1999. *Methods for determining bactericidal activity of antimicrobial agents : approved guideline*, National Committee for Clinical Laboratory Standards, Wayne, PA.
47. Ceri H, Olson ME, Stremick C, Read RR, Morck D, Buret A. 1999. The Calgary Biofilm Device: new technology for rapid determination of antibiotic susceptibilities of bacterial biofilms. *J. Clin. Microbiol.* **37:** 1771–1776. https://doi.org/10.1128/JCM.37.6.1771-1776.1999.
48. Macia MD, Rojo-Molinero E, Oliver A. 2014. Antimicrobial susceptibility testing in biofilm-growing bacteria. *Clin. Microbiol. Infect.* **20:** 981–990. https://doi.org/10.1111/1469-0691.12651.
49. Goldman MJ, Anderson GM, Stolzenberg ED, Kari UP, Zasloff M, Wilson JM. 1997. Human beta-defensin-1 is a salt-sensitive antibiotic in lung that is inactivated in cystic fibrosis. *Cell.* **88:** 553–560. https://doi.org/10.1016/s0092-8674(00)81895-4.
50. Ma T, Liu Y, Yu B, Sun X, Yao H, Hao C*, et al.* 2025. DRAMP 4.0: an open-access data repository dedicated to the clinical translation of antimicrobial peptides. *Nucleic Acids Res.* **53:** D403–D410. https://doi.org/10.1093/nar/gkae1046.
51. Decker AP, Su Y, Mishra B, Verma A, Lushnikova T, Xie J*, et al.* 2023. Peptide Stability Is Important but Not a General Requirement for Antimicrobial and Antibiofilm Activity *In Vitro* and *In Vivo*. *Mol. Pharm.* **20:** 738–749. https://doi.org/10.1021/acs.molpharmaceut.2c00918.
52. Andersson DI, Hughes D, Kubicek-Sutherland JZ. 2016. Mechanisms and consequences of bacterial resistance to antimicrobial peptides. *Drug Resist. Updat.* **26:** 43–57. https://doi.org/10.1016/j.drup.2016.04.002.
53. Joo HS, Fu CI, Otto M. 2016. Bacterial strategies of resistance to antimicrobial peptides. *Philos. Trans. R. Soc. Lond B Biol. Sci.* **371:** 20150292. https://doi.org/10.1098/rstb.2015.0292.
54. Odds FC. 2003. Synergy, antagonism, and what the chequerboard puts between them. *J. Antimicrob. Chemother.* **52:** 1. https://doi.org/10.1093/jac/dkg301.
55. Fatsis-Kavalopoulos N, Sanchez-Hevia DL, Andersson DI. 2024. Beyond the FIC index: the extended information from fractional inhibitory concentrations (FICs). *J. Antimicrob. Chemother.* **79:** 2394–2396. https://doi.org/10.1093/jac/dkae233.
56. Taheri-Araghi S. 2024. Synergistic action of antimicrobial peptides and antibiotics: current understanding and future directions. *Front. Microbiol.* **15:** 1390765. https://doi.org/10.3389/fmicb.2024.1390765.
